# Cutaneous Metastasis from Internal Malignancies: The Revealing Role of the Skin

**DOI:** 10.3390/cancers15174351

**Published:** 2023-08-31

**Authors:** Gerardo Cazzato, Anna Colagrande, Eliano Cascardi, Giuseppe Ingravallo

**Affiliations:** 1 Section of Molecular Pathology, Department of Precision and Regenerative Medicine and Ionian Area (DiMePRe-J), University of Bari “Aldo Moro”, 70124 Bari, Italy; anna.colagrande@gmail.com (A.C.); giuseppe.ingravallo@uniba.it (G.I.); 2 Department of Medical Sciences, University of Turin, 10124 Torino, Italy; eliano20@hotmail.it; 3 Pathology Unit, FPO-IRCCS Candiolo Cancer Institute, Str. Provinciale 142 km 3.95, 10060 Candiolo, Italy

Skin represents the heaviest organ of the human body, covering a surface area of 1.5 to 2 square metres, varying in thickness from 0.5 to 3 mm and weighing approximately 10 kg (in adult humans) [1]. Although primitive neoplastic lesions, such as Basal Cell Carcinoma (BCC), Squamous Cell Carcinoma (SCC), Malignant Melanoma (MM) and Merkel Cell Carcinoma (MCC), are common, the skin is also often the site of secondary localisation of neoplasms of different nature, provenance and stage, which may become noticeable on the skin generally after they have already been diagnosed. However, in rare cases, the skin may show the first sign of an internal malignancy, which is investigated and discovered at a later stage [2]. According to the data reported in [2], the percentage of skin metastases from primary malignancies is as follows: melanoma has a 45% chance of developing cutaneous metastasis (but only 15–20% of melanomas metastasize, so the overall chance of skin metastasis is about 7–10%); 30% for breast cancer; 20% (specifically 12%) for nasal sinus cancers; 16% for larynx cancer; and 12% for oral cavity cancer. Therefore, it is important to consider that the age and sex of the patients influence the detection of different types of skin metastases originating from different organs. In males under 40 years of age, MM, colorectal carcinoma and lung carcinoma occur in decreasing order of frequency, whereas males above 40 years of age are more likely to have lung carcinoma, colorectal carcinoma, squamous cell carcinoma of the oral cavity and, finally, MM. In females under 40 years of age, the main organs of origin for skin metastases are breast carcinoma, colorectal carcinoma and ovarian carcinoma, whereas women over 40 years of age are more likely to have lesions from breast carcinoma, colorectal carcinoma, lung carcinoma, ovarian carcinoma and MM [2]. In 2012, an interesting and comprehensive study conducted by Alcaraz I. et al. analysed the types of skin metastases from internal malignancies, focusing on the clinical and histopathological characteristics of the lesions. The most frequent type of metastatic carcinoma affecting the skin originates from the breast, with more frequent localisation on the chest wall and abdomen, as well as on the extremities and the head/neck region [3]. The authors emphasised the different clinical and histopathological features that breast carcinoma can adopt in a metastatic setting. Second, lung carcinoma (the first cause of metastatic lesions in males over 40 years of age) may manifest earlier in the metastatic setting before the primary tumour is diagnosed, an aspect emphasised in one study [4]. In terms of histotype, approximately 50% of the cases originate from non-small cell carcinomas, whereas 30% of the cases originate from small-cell lung carcinomas [5]. On the other hand, various other organs can be the origin of cutaneous metastases, such as the gastrointestinal system (colorectal, stomach, liver, gallbladder, pancreas, esophagus and oral cavity).

In this Special Issue (SI), we aim to showcase contributions that focus on case reports, case series, original articles or reviews centred on skin metastases from internal malignancies, emphasising how multidisciplinary and holistic knowledge of human pathology is indispensable for correct diagnosis and therapeutic and prognostic framing.
